# Excited State Dynamics of 8-Vinyldeoxyguanosine in Aqueous Solution Studied by Time-Resolved Fluorescence Spectroscopy and Quantum Mechanical Calculations

**DOI:** 10.3390/molecules25040824

**Published:** 2020-02-13

**Authors:** Lara Martinez-Fernandez, Thomas Gustavsson, Ulf Diederichsen, Roberto Improta

**Affiliations:** 1 Departamento de Química, Facultad de Ciencias and IADCHEM (Institute for Advanced Research in Chemistry) Universidad Autónoma de Madrid, Cantoblanco, 28049 Madrid, Spain; lara.martinez@uam.es; 2 Université Paris-Saclay, CEA, CNRS, LIDYL, 91191 Gif-sur-Yvette, France; 3 Univ Goettingen, Inst Organ & Biomol Chem, Tammannstr 2, D-37077 Goettingen, Germany; udieder@gwdg.de; 4 Istituto di Biostrutture e Bioimmagini, CNR, Via Mezzocannone 16, I-80134 Napoli, Italy

**Keywords:** DNA, solvent effect, TD-DFT, fluorescent probe

## Abstract

The fluorescent base guanine analog, 8-vinyl-deoxyguanosine (8vdG), is studied in solution using a combination of optical spectroscopies, notably femtosecond fluorescence upconversion and quantum chemical calculations, based on time-dependent density functional theory (TD-DFT) and including solvent effect by using a mixed discrete-continuum model. In all investigated solvents, the fluorescence is very long lived (3–4 ns), emanating from a stable excited state minimum with pronounced intramolecular charge-transfer character. The main non-radiative decay channel features a sizeable energy barrier and it is affected by the polarity and the H-bonding properties of the solvent. Calculations provide a picture of dynamical solvation effects fully consistent with the experimental results and show that the photophysical properties of 8vdG are modulated by the orientation of the vinyl group with respect to the purine ring, which in turn depends on the solvent. These findings may have importance for the understanding of the fluorescence properties of 8vdG when incorporated in a DNA helix.

## 1. Introduction

Steady-state and time resolved fluorescence spectroscopy has greatly contributed to the understanding of the structure and dynamics of various biological systems [1], both in the ground and in the electronic excited states. However, since many of the most important biomolecules are not highly fluorescent, a huge effort is currently consecrated to the synthesis of new fluorescent analogs [2]. This is particularly the case for DNA, where the natural building blocks, the nucleobases, are characterized by very low fluorescence quantum yields, of the order of 10^−4^ [3,4], rendering their use for analytical purposes difficult. Therefore, active research has been dedicated to developing fluorescent nucleobase analogs that are structurally as close as possible to the natural bases in order to be incorporated in the DNA helix without causing any major perturbations [2]. Ideally, the base analogs should respect the hydrogen-bonded structures of the natural base pairs and be isomorph, that is, having similar dimensions. They should also have absorption spectra that are well distinguished, that is, red-shifted, with regards to the natural bases. This is important for two reasons; (i) it allows selective excitation and detection and (ii) the exciton coupling with the surrounding natural nucleobases is absent [5]. Under these conditions, the fluorescence properties of the base analog monitor the local environment. Actually, most of the information gained by using fluorescent probes is obtained by monitoring the quenching of the fluorescence. More precisely, base analogs may be highly fluorescent in homogeneous solution, while their fluorescence is strongly quenched when incorporated in the DNA helix. This quenching may in turn be attenuated when the helix undergoes structural changes or interacts with other biomolecules, increasing fluorescence intensity. Examples of biological processes monitored with the aid of fluorescent nucleobase analogues are DNA-protein interactions such as DNA methyltransferase, polymerase, endonuclease and uracil glycosylate [6].

In this context, 2-aminopurine (2AP) is perhaps the most well-known fluorescent base analog, being isomorph with adenine and forming base-pairs with pyrimidines. When incorporated in oligonucleotides, 2AP emission is strongly quenched [7,8], supposedly due to electron transfer from an adjacent purine base. Since the degree of fluorescence quenching depends on the local environment, 2AP fluorescence is used to study conformational changes of the DNA helix [9]. However, 2AP cannot be considered an ideal fluorescent probe, since the anti-conformer does not have the same base-pairing configuration as adenine and it can give a mismatch with cytosine [10,11,12]. NMR measurement on model duplexes have shown that the structural changes induced by 2AP are relatively minor and local but that the helix opening dynamics may be strongly affected [13].

In contrast to 2AP, substitution of purines with a vinyl moiety at the 8-position does not affect the hydrogen bonding properties but increases dramatically the fluorescence yield. It should be noted that the additional vinyl substituent at the 8-position does not affect base pairing with thymine in duplex DNA [14,15]. Like 2AP, when incorporated in oligonucleotides, 8-vinyladenosine (8vA) fluorescence is strongly quenched and the amount of quenching depends strongly on the local sequence [15,16,17]. Different explanations for the high fluorescence yield of 8vA have been proposed but no final picture has yet been obtained [18,19]. It is clear that the highly efficient internal conversion mechanism active in adenosine is inhibited but the decay pathways have not been mapped and the molecular mechanism underlying the high fluorescence QY not yet assessed. The excited state properties of 8vA have also been studied by Stark spectroscopy and cyclic voltammetry [19,20].

The vinyl group substitution has also been applied to guanosine, resulting in a highly fluorescent analogue, 8-vinylguanosine (8vG) [21,22,23], which shows very promising properties. It respects the same core structure as guanosine, which means that it forms the same hydrogen bonds when base pairing with cytidine. Moreover, it can adapt both *syn* and *anti*-conformations which makes it very interesting for studying various quadruplex structures. Finally, while being highly fluorescent, its emission properties are very sensitive to the local environment, such as the surrounding helix or quadruplex structure. For example, when 8-vinyl-2′-deoxyguanosine (8vdG) is incorporated in a human telomeric sequence, its emission is three-fold higher in the presence of NaCl than KCl, showing its strong topological differentiation [21].

Recently, Kochman et al. published a theoretical study of the photophysics of 8vG in the gas phase, including the bulk solvent effect by single point calculations (using the ADC(2) method) on the gas phase structures [24]. They found two close-lying excited ππ* states with high oscillator strength in the Franck-Condon (FC) region, separated by less than 0.5 eV. The lowest excited state has substantial intramolecular charge transfer (ICT) character while the second one is localized on the guanine moiety. After excited state optimization they found that the only emissive electronic state is the ICT ππ* state. A minor radiationless deactivation process involves departure from the minimum of the ICT state towards a region where it adopts a 9H-guanine like structure enabling the internal conversion to the ground state. Including solvent effect by a continuum model and single point calculations on the gas-phase geometries, they predicted that the stability of the minimum and, therefore, the excited state lifetime depend on the solvent.

Intrigued by these interesting results and striving to get a more accurate understanding of the excited state relaxation processes occurring in 8vdG in solution, we here investigate its photophysics in greater detail, combining steady-state and time-resolved optical spectroscopies as well as further quantum mechanical calculations. In detail, the photophysical properties (steady-state absorption and fluorescence spectra, fluorescence lifetimes) of 8vdG are thoroughly studied experimentally in water, methanol, ethanol, acetonitrile and THF—five solvents characterized by different polarities and proticities. Moreover, we present the first study of 8vdG in aqueous solution using femtosecond fluorescence spectroscopy, providing new information on the spectral relaxation and the anisotropy. The ensemble of these new experimental data highlights the large stabilizing effect induced by the vinyl group. In parallel, we extend the theoretical analysis of Kochman et al. [24], focusing on solvent effect, discriminating the role of bulk solvent effect and solute-solvent hydrogen bonds and trying to assess the importance of dynamical solvation effects. Furthermore, the role played by the sugar conformation (*syn*/*anti*) is investigated and the results obtained for the nucleoside are compared with those relative to a simpler system, where the sugar is modeled by a methyl group. On these grounds, we obtain interesting insights into the subtle interplay between solvent and vibronic effects; the latter related to the interaction between the π system of the purine ring and the vinyl moiety. 

The experimental and computational findings are in good agreement and provide a consistent picture of the 8vdG photophysics in different solvents and of its perspectives as DNA fluorescent probe. We show that the photophysical properties of 8vdG depend strongly on the orientation of the vinyl group, providing a potential microscopic probe of the local structure.

## 2. Material and Methods

### 2.1. Experimental Details

#### 2.1.1. Steady-Stated Spectroscopy

8-vinyl-2′-deoxyguanosine (8vdG) was synthesized as described in refs. [21,22,23]. For the solutions, UV Grade methanol (MeOH), ethanol (EtOH), acetonitrile (MeCN) and tetrahydrofuran (THF) from Sigma-Aldrich (St. Louis, MO, USA) were used. For the aqueous solution, ultrapure water from a Millipore Milli-Q system (Merck Millipore, Merck KGaA, Darmstadt, Germany) was used.

Steady-state absorption and emission spectra were recorded with a double-beam UV-visible Lambda 900 spectrophotometer (PerkinElmer Inc., Hopkinton, MA, USA) and a SPEX Fluorolog 3 spectrofluorometer (Horiba Jobin Yvon, Chilly Mazarin, France) in 1 mm and 1 cm optical path cells, respectively. The fluorescence spectra were corrected for the weak residual fluorescence of the neat solvent and the wavelength-dependent response of the instrument.

#### 2.1.2. Time-Resolved Fluorescence Spectroscopy

Time-resolved fluorescence measurements were performed using the time-correlated single photon counting (TCSPC) and fluorescence upconversion (FU) techniques. The same excitation source was used for the two kinds of experiments: the third harmonic (267 nm) of a mode-locked Ti-Sapphire laser (MIRA 900, Coherent Inc., Santa Clara, CA, USA), delivering ~120 fs pulses at a repetition rate of 76 and 4.75 MHz for FU and TCSPC, respectively (in the latter case set by a pulse-picker).

For the TCSPC experiments, solutions were contained in a 10 mm × 10 mm quartz cell and continuously stirred. The fluorescence was collected at right angle and focused onto the entrance slit of a small HR250 monochromator (Horiba Jobin Yvon, 91,380 Chilly Mazarin, France) using off-axis parabolic mirrors. In order to cut the laser excitation light, a WG295 filter (Schott AG, Mainz, Germany) was placed in front of the slit. Moreover, a Glan-Thompson polarizer (ThorLabs, Maisons-Laffitte, France) ensured that only the vertical component of the fluorescence was detected. The selected fluorescence was detected by a R1564U microchannel plate (Hamamatsu, Massy, France) in single photon counting mode connected to a SP-630 PC card (Becker & Hickl GmbH, Berlin, Germany). The laser pulse synchronization was done with a rapid photodiode (ThorLabs, Maisons-Laffitte, France) [25].

In the FU experiments, solutions were flowed through a 1 mm quartz cell. The average excitation power was 60 mW. The fluorescence was collected with two parabolic mirrors and mixed with the residual 800 nm gate beam in a 0.5-mm BBO crystal (type I, only sensitive to the vertical component). The gate beam passed through a mechanical delay stage allowing the control of the relative time delay. The up-converted signal was then dispersed (spectral resolution c.a. 5 nm) in a double-grating SPEX 1680 monochromator (Horiba Jobin Yvon, Chilly Mazarin, France) and detected by a 1527P photomultiplier (Hamamatsu, Massy, France) connected to a SR400 photon counter (Stanford Research Systems Inc., Sunnyvale, CA, USA) [26].

For both TCSPC and FU, the decays at different emission wavelengths were made either at the magic angle (54.7°) or for parallel (*I_par_*) and perpendicular (*I_perp_*) excitation/emission configurations by controlling the polarization of the exciting beam with a half-wave plate in FU (Jean Fichou, Fresnes, France) or a Fresnel rhomb in TCSPC (EKSMA Optics, UAB, LT-08412 Vilnius, Lithuania). The excitation energies under parallel and perpendicular conditions were identical, giving a G factor of 1.

In the latter case, the total fluorescence was calculated as *F(t) = I_par_(t) + 2I_perp_(t)*. The fluorescence anisotropy was calculated as *r(t) = (I_par_(t) − I_perp_(t))/F(t)*.

In order to evaluate the characteristic times involved, we performed a merged nonlinear fitting/deconvolution process using a multi-exponential model function *i(t)* convoluted by the instrument response function (irf), *I(t)* ∝ *i(t)* ⊗ *irf(t)*. In the case of TCSPC, the irf was determined to be 60 ps (fwhm) using the Raman line in water at 295 nm. In the case of FU, an analytical Gaussian function with a full width half maximum (fwhm) of 350 fs was used as irf. We judge that the time resolution is about 50 ps and 100 fs for TCSPC and FU data respectively after deconvolution.

Time-resolved fluorescence spectra were reconstructed following the procedure outlined by Maroncelli and Fleming [27]. The time integral of each model function i(t,λ) for a given wavelength is scaled with regards to the corresponding intensity in the corrected steady-state fluorescence spectrum $I^{SS}\left(\lambda \right)$ according to Equation (1).

(1) $$\int_{0}^{\infty }i\left(t,\lambda \right)dt=\int_{0}^{\infty }c\left(\lambda \right)\sum_{i}a_{i}^{\lambda }e^{-t/\tau_{i}^{\lambda }}dt=c\left(\lambda \right)\left(\sum_{i}a_{i}^{\lambda }\tau_{i}^{\lambda }\right)=I^{SS}\left(\lambda \right).$$

In this equation, $a_{i}^{\lambda }$ and $\tau_{i}^{\lambda }$ are the pre-exponential factors and the time-constants defining the multi-exponential model function *i(t)*. The scaling factor c(λ) is simply obtained from Equation (1) as $c\left(\lambda \right)=I^{SS}\left(\lambda \right)/\sum_{i}a_{i}^{\lambda }\tau_{i}^{\lambda }$ and the corresponding scaled spectral points at a given wavelength λ for a given delay time *t* are given as $i\left(t,\lambda \right)=c\left(\lambda \right)\sum_{i}a_{i}^{\lambda }e^{-t/\tau_{i}^{\lambda }}$.

Plotting the scaled model functions for a given time delay as a function of the wavelength gives the corrected time-resolved fluorescence spectrum.

### 2.2. Computational Details

#### 2.2.1. Models

**Scheme 1:**
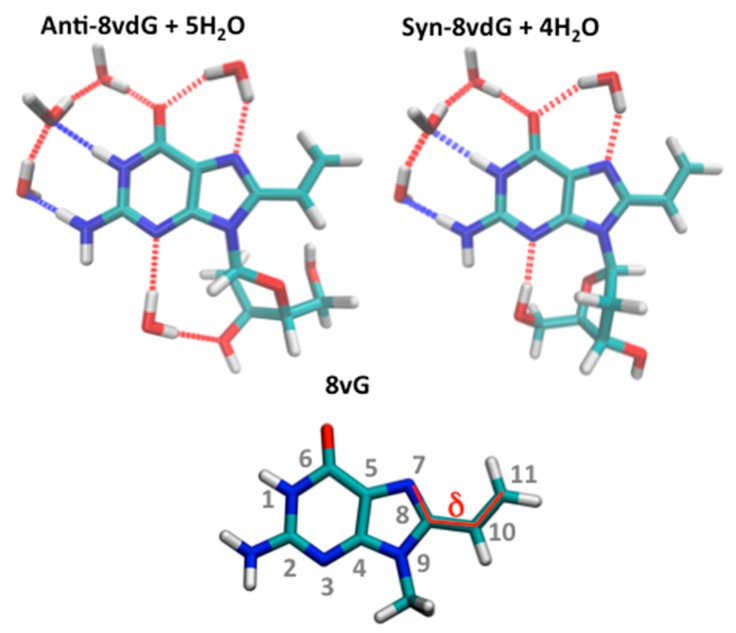
Schematic drawing of the different computational models used in this study.

8vdG and its methyl derivative 9-methyl-8-vinyl-guanine (8vG) were studied in this work (Scheme 1). Two different conformers (*syn* and *anti*), involving different rotations around the glycosidic bond (Scheme 1), were considered for 8vdG. 

#### 2.2.2. Methods

Density Functional Theory (DFT) was used for the optimization of the different ground state (GS) minima, whereas with its Time Dependent version (TD), we characterized the excited states, that is, Vertical Absorption Energies (VAE) and Potential Energy Surfaces (PES). Unless specified, the CAM-B3LYP [28] functional was selected throughout this study, due to its good performance describing the photophysics of nucleobases [29,30,31]. The calculations of 8vdG were performed with the 6-31G(d) basis set; however, larger bases (6-31+G(d,p)) were also considered for 8vG (see Supplementary Materials). Due to the limitations of TD-DFT in the description of the Conical Intersections (CI) with the ground state [32,33], no attempt has been made to locate their exact position. On the other hand, we have verified that the structure of the crossing region with S_0_ located at the ADC(2) level [24] was retrieved at the TD-CAM-B3LYP level, by performing a relaxed scan associated to the out-of-plane motion of the NH_2_ moiety and considering the most stable structure with energy gap with S_0_ < 0.5 eV as representative of the crossing region.

#### 2.2.3. Solvation Models

Acetonitrile (MeCN) and Water (H_2_O) were modelled using the implicit Polarizable Continuum Model (PCM) [34,35]. However, considering the presence of H-bonds between solvent molecules and 8vdG can be essential for a correct description of several properties, as for instance, the *syn*-*anti* equilibrium [36]. We have introduced explicit water molecules in our calculations using, then, a hybrid implicit/explicit model (PCM + H_2_O). Different number of water molecules (4 or 5) was considered depending on the selected system (Scheme 1). Overall, the Linear Response (LR-PCM) version of the PCM was used but at specific points of the PES we have resorted to State Specific (SS-PCM) [37,38] calculations since it provides a more accurate estimate of electronic transitions with significant CT character. In this latter case, calculations have been performed bot in the non-equilibrium (NE) and equilibrium (E) time-regimes. The former provides that only solvent electronic polarization, fast solvent degrees of freedom, is in equilibrium with the excited state electron density of the solute, while the slow solvent response (related to the solvent nuclear rearrangements) remain in equilibrium with the ground state electron density. In the latter regime both slow and fast solvent polarization is in equilibrium with the electron density of the state of interest.

All the calculations were performed using the Gaussian09 program [39].

Radiative time constants were calculated using Equation (2).

(2) $${}^{Theor}\tau_{R}=\frac{1}{k_{R}}\;k_{R}=\frac{4}{3}\frac{{\mathrm{VEE}}^{3}}{c^{3}}\mu^{2},$$

where c is the speed of light, VEE are the emission energies and μ the transition dipole moment. The fluorescence lifetime ($\tau_{F}$) can be estimated by multiplying the radiative time constant by the (experimental) fluorescence quantum yield.

## 3. Results

### 3.1. Steady-State Spectroscopy

**Figure 1:**
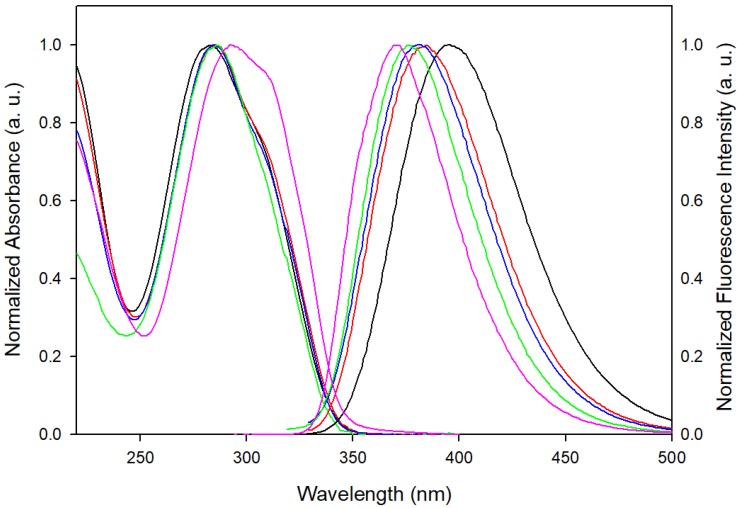
Normalized steady-state absorption and fluorescence spectra of 8-vinyl-deoxyguanosine in H_2_O (black), MeOH (red), EtOH (blue), MeCN (green) and THF (pink).

Normalized steady-state absorption and fluorescence spectra of 8vdG in H_2_O, MeOH, EtOH, MeCN and THF are presented in Figure 1. The absorption and emission maxima, in both wavelength and wavenumber, are given in Table 1.

The absorption spectra are characterized by a first structured band peaking around 285 nm with a weak “shoulder” around 300 nm. The position and the shape of this first absorption band depends little on the solvent, with the exception of the low-polar and aprotic THF, where the maximum red-shifts to 293 nm. In this latter solvent the long wavelength “shoulder” becomes more prominent, being almost as intense as the maximum. A second, much stronger band peaks below 200 nm in all the solvent but its analysis falls outside the scope of the present study. 

In Figure S1 we show the absorption spectra after transformation to an energy scale. In all solvents, the first absorption band clearly shows a complex structured shape indicating several overlapping transitions. Calculating the second derivative indicates that there are two main transitions involved, located around 32,000 and 35,000 cm^−1^ (Figure S2).

In order to get a better description of the two transitions, the absorption spectra were model-fitted by a sum of two lognormal functions (Figure S3). Except for the high energy wing, the experimental spectra are very well reproduced. The first absorption band can thus be decomposed into two transitions around 32,000 and 35,000 cm^−1^ with an intensity ratio of about 1 to 3. The fitted peak positions for the two lowest transitions in the five solvents are given in Table S1. It can be seen that excitation at 267 nm nearly exclusively populates the higher transition. 

The fluorescence bands (Figure 1) are broad, unstructured and peak between 370 and 400 nm. Going from the low-polar and aprotic THF to the more polar and protic water there is a marked red-shift of the fluorescence spectra. This red-shift is not simply dependent on the polarity (see Table S1) but solute/solvent hydrogen bonds clearly play a role. For example, in aprotic MeCN the maximum is less red-shifted than in the two protic alcohols, despite the larger dielectric constant of the former solvent. However, a more quantitative solvatochromic analysis is outside the scope of the present work.

### 3.2. Time Resolved Fluorescence Spectroscopy

**Figure 2:**
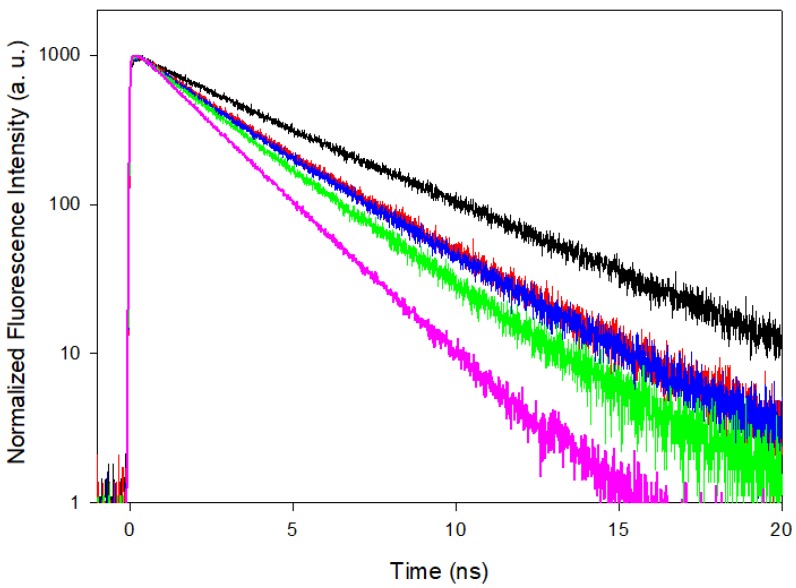
Fluorescence decays of aerated solutions of 8vdG in H_2_O (black), MeOH (red), EtOH (blue), MeCN (green) and THF (pink). The excitation wavelength was 267 nm, detection at the fluorescence maximum around 375 nm.

Fluorescence decays on the nanosecond timescale of aerated solutions of 8vdG in H_2_O, MeOH, EtOH, MeCN and THF were recorded at the fluorescence maximum after excitation at 267 nm, see Figure 2.

The decays are strictly mono-exponential with characteristic time constants ranging from 2.1 ns in THF to 4.35 ns in H_2_O, see Table 2. There are no indications of any faster components at early times. The fluorescence lifetimes increase with increasing solvent polarity and hydrogen-bonding character. Assuming that the excited state properties are similar for 8vdG and 8vG, we can estimate the radiative lifetime of 8vG in water using the published value for the fluorescence quantum yield of, Φ_F_ = 0.72 ± 0.3 [21]. The calculated value is 6 ns, corresponding to an allowed transition.

**Figure 3:**
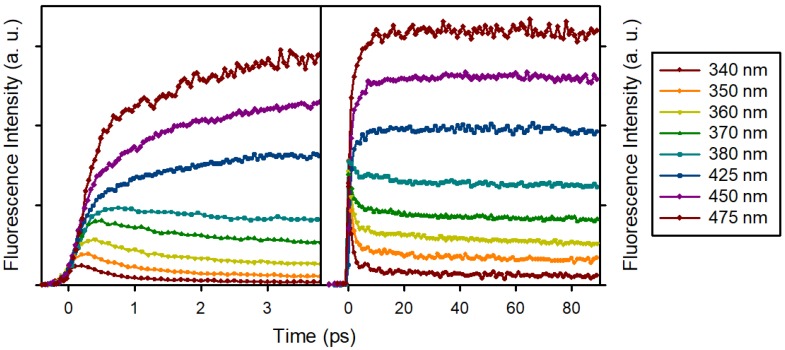
Femtosecond fluorescence decays of 8vdG in H_2_O after excitation at 267 nm. Successive traces at 340, 350, 360, 370, 380, 425, 450 and 475 nm are shown from the bottom to the top. The curves are not proportional to the measured intensity but scaled for clarity.

Femtosecond fluorescence upconversion traces of an aerated solution of 8vdG in H_2_O were recorded between 330 and 550 nm in 5 and 100 ps time windows. Selected total fluorescence decays F(t) are shown in Figure 3.

The fluorescence decays are clearly bi-modal with a fast picosecond evolution followed by a practically constant level on the studied timescale. It seems reasonable to assume that the longer component corresponds to the 4.35 ns fluorescence lifetime described above. 

The decays were thus fitted using a tri-exponential model function with two free-floating terms and a fixed 4.35 ns component. The fitted time constants are given in Table 2.

It is important to note that the ps components correspond to a fast decay on the blue side and a fast rise on the red side by which is characteristic for a dynamic spectral shift. 

Interestingly, the ps evolution is composed by an ultrafast component of about 1 ps and a second one on the order of tens of ps. 

In order to better characterize the spectral shift, time-resolved fluorescence spectra were reconstructed from the fixed wavelength decays using the standard procedure described in the Material and Methods section above (Equation (1)) and the fitted time constants from Table 2.

**Figure 4:**
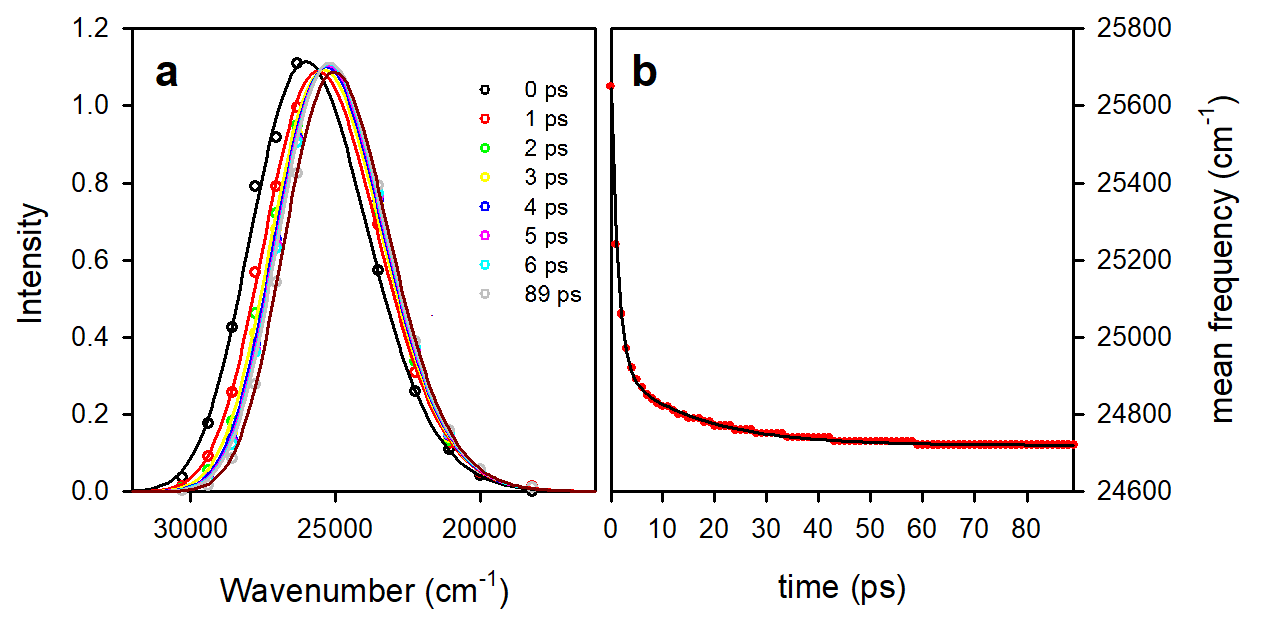
(**a**) Experimental reconstructed and fitted time-resolved fluorescence spectra of 8vdG in H_2_O at chosen delay times, (**b**) the mean frequency of the fitted fluorescence spectra of 8vdG in H_2_O as a function of time.

The time-resolved fluorescence spectra thus obtained were put on an energy scale by multiplying by *λ*^2^ and were subsequently fitted with a simplified lognormal function for each delay time (see Supplementary Materials and Figure S4). This allows to calculate such parameters as the peak and the mean position as well as the width of the fluorescence band and to analyze their time-dependence. Examples of experimental and fitted spectra are shown in Figure 4a.

On an energy scale, the fluorescence spectra are close to symmetrical Gaussians. Indeed, the shape, width and asymmetry changes little in time. More interestingly, there is a very rapid red-shift during the first picosecond followed by a slower relaxation that extends to several tens of picoseconds. This is illustrated in Figure 4b, where the mean frequency as a function of time is shown. The time dependence can be well described by a bi-exponential model function with 1.3 and 15.5 ps time constants. If the total shift is dominated by the fast component, the slower shift still accounts for more than 20%. 

While the ultrafast component can be interpreted as due to water solvation dynamics which is known to occur on a sub-ps to ps time scale [40], the second component is too slow for this process. Therefore, we assign this slower spectral relaxation to another process, likely connected to a conformational change, as discussed below.

#### Fluorescence Anisotropy Decays

**Figure 5:**
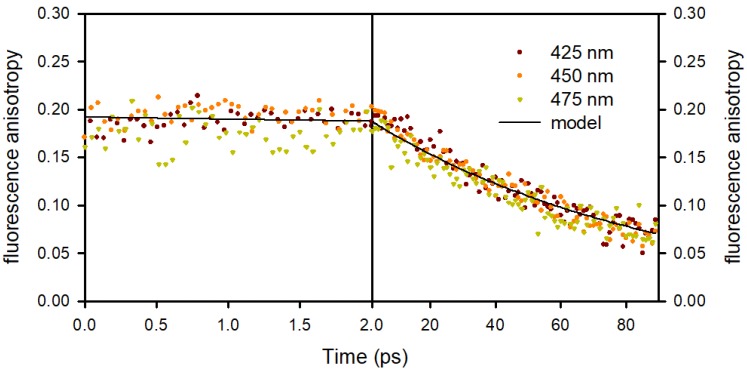
Fluorescence anisotropies of 8vdG in water at various wavelengths after excitation at 367 nm.

The fluorescence anisotropies at various wavelengths are shown in Figure 5. A simple mono-exponential model gives a zero time value of r_0_ = 0.19 ± 0.01 and a decay time of about 90 ps. The fitted curve is indicated as a solid line in Figure 5. 

A similarly low zero time value r_0_ was also observed for dGMP [41] and can be readily explained by the fact that excitation at 267 nm populates a higher excited state (S_2_), which subsequently transfers to the lowest excited state (S_1_) inducing a change in the orientation of the transition dipole moment [42]. No clear wavelength dependence is observed, in line with the emission coming only from S_1_.

### 3.3. Computational Results 

#### 3.3.1. Ground State Equilibria

In the ground state (GS), 8vdG could eventually exist in two different forms, *syn* and *anti*, respectively (Scheme 1), depending on the rotation about the glycosidic bond. The energy difference between the two conformers is not large (always <0.2 eV, Table S2). In MeCN, the *syn* conformer is slightly more favored due to the formation of a strong intramolecular hydrogen bond. In water, the anti-conformer is marginally more stable (by <0.2 kcal/mol), due to the effect of the solute-solvent hydrogen bonds. Considering that only a dedicated study including entropic effects in reliable way can assess the *syn*/*anti* conformational equilibrium of isolated nucleosides in solution, our calculations suggest that both of them are present, in agreement with the experimental indications on other purine bases [43,44].

Geometry optimizations indicate that in the GS of 8vG the vinyl group is almost coplanar with the purine ring both in MeCN (δ = C_11_-C_10_-C_8_-N_7_ = 0°, see Scheme 1) and in H_2_O (δ = −3°), allowing for a maximization of the electronic interactions between the two moieties. Constrained optimizations along δ dihedral show that the rotational energy barrier for an isomerization of the vinyl group, measured as the energy difference between the GS minimum and a structure with perpendicular arrangement of the vinyl (δ = 90°, corresponding to a saddle point), which would not allow any meaningful electronic interactions with the guanine ring, is not very large, that is, 4.5 kcal/mol in H_2_O and 5.1 kcal/mol in MeCN, at the CAM-B3LYP 6-31G(d) level (Table S3). Furthermore, the energy difference between the GS minimum and a conformation with δ = 45° (i.e., roughly intermediate between the minimum and the maximum) is significantly lower (<2 kcal/mol) in both solvents (Table S3 and Figure S5), suggesting that at room temperature significant departure from a perfectly planar arrangement of the vinyl group are possible. When the sugar is included in our computational model (8vdG), its steric hindrances decreases the relative stability of the planar structures, being the dihedral values at the GS minimum, δ = −17° and −34° respectively for the *syn*- and *anti*-conformers in water (Figure S5). The computed rotational energy barrier around δ is thus lower for 8vdG than for 8vG, with the single exception of the anti-tautomer in MeCN (Table S3).

#### 3.3.2. The Excited States in the Franck-Condon Region.

The Vertical Absorption Energies between the ground state and the lowest energy excited states were computed at the TD-CAM-B3LYP level for the three compounds 8vG, *syn*-8vdG, *anti*-8vdG in three solvents THF, MeCN and H_2_O using two computational models (LR-PCM, SS-PCM). The resulting transition energies are collected in Table 3 and Table S4.

Three main bright excited states are located within 1 eV, their interaction being modulated by the value of the dihedral angle δ. These three states can be more easily identified for δ = 90°, when the vinyl moiety cannot interact with the guanine ring system (Table S4). For this latter geometry there are two excited states that almost coincide with the two lowest energy bright states of Guanine (Table S5): the lowest energy one, S1, less intense, can be described as a HOMO → LUMO excitation, usually labeled as La and ~0.5 eV blue-shifted, the more intense transition which can be described as a HOMO → LUMO + 1 excitation, usually labeled as L_b_ [42]. When δ decreases and thus the π systems of the two moieties can interact, the two lowest energy excited states start mixing significantly with the vinyl moiety. For δ = 45°, the two lowest bright states are significantly red-shifted with respect to Guanine and have similar intensities. Finally, in the global minimum (δ close to 0), the lowest energy transition is predicted to be significantly more intense than S_2_ and both transitions experience an additional red-shift. The analysis of the excited–ground state density difference in the FC point (Figure S6) indicates that for all systems in all solvents, the density S_1_–S_0_ is delocalized between the guanine and the vinyl group and that this excited state has a significant intramolecular charge transfer (ICT) character from the guanine moiety to the vinyl group. This conclusion, in agreement with previous results [24], is confirmed by Mulliken Population analysis, showing that the S_1_ transition involves the G → vinyl CT of 0.16–0.20 a.u. (Figure S6). 

On the contrary, the S_2_ state is mainly localized on the guanine nucleobase (π(G)-π*(G)), being the counterpart to the well-known ππ*(L_b_) excited state found in the canonical base. The computed CT character for all the systems in all solvents associated to the S_2_ excited state is almost negligible (0.07–0.02 a.u).

In the three solvents, both S_1_ and S_2_ show high oscillator strength and are separated by ~0.4 eV, in good agreement with the experimental absorption spectrum, where the lowest absorption band show the signature of two bright transitions separated by ~0.5 eV (4000 cm^−1^, see Table S1). On the other hand, our calculations predict that the lowest energy transition is much more intense than the second one, contrary to the experimental results. According to the considerations made above, it seems that our calculations overestimate the electronic interaction between the vinyl group and the guanine ring system. This could be due to the adopted electronic methods, but, on the other hand, also ADC(2) calculations provide a similar description of the relative intensity of S_1_ and S_2_ [24]. Interestingly, when the vinyl moiety does not interact with the ring (δ = 90°), the relative intensity of the two peaks is reverted, being much more similar to the experimental one. It is thus possible that, because of the low energy barrier for rotation around δ, the ‘effective’ degree of planarity of 8vG is smaller than that found in the minimum. Actually, in water the lowest absorption peak of 8vdG is at ~4.3 eV (286 nm), that is, red-shifted by ~0.4 eV with respect to that of Guanine at ~4.7 eV (266 nm). According to our calculations, such a blue-shift corresponds to a value of intermediate between 0° (predicted shift 0.6 eV) and 45° (predicted shift 0.3 eV). 

Our calculations indicate that the absorption spectra exhibit a very small dependence on the solvent. More precisely, for 8vG, whose absorption spectrum does not depend on the orientation of the glycose ring, the results obtained in MeCN and THF are very similar, the differences being well within the expected accuracy of excited state calculations in solution and for an analysis based only of vertical transitions. A small red-shift is instead predicted for 8vG when going from THF to water, which is contrary to the experimental indications but in line with what is expected of an electronic transition with partial CT character. A similar qualitative picture is found for the *syn*-conformer of 8vdG, though, in this case, the red-shift is smaller. For the anti-conformer of 8vdG, a substantial blue-shift (>800 cm^−1^) is instead predicted when going from THF or MeCN to water, both at the LR-PCM and the SS-PCM level. The trend found for this latter compound can be explained by the larger dihedral angle δ in the S_0_ minimum of anti 8VdG in water, due to the steric hindrances between the guanine ring, the vinyl moiety and the water molecules. Interestingly, if we consider that the *syn* conformer is relatively more stable in THF and the anti-conformer in water, we would predict a fairly large blue-shift for both excited states, in line with the experimental indications.

The CT character is higher in MeCN (8vG-0.20: 8dvG-0.18) compared to H_2_O (0.16) and, in the same solvent, is higher when the sugar is not present. These differences are likely also connected to the different degree of planarization of the system, the CT character increasing for planar geometries. 

#### 3.3.3. Excited State Optimization and Emission 

**Figure 6:**
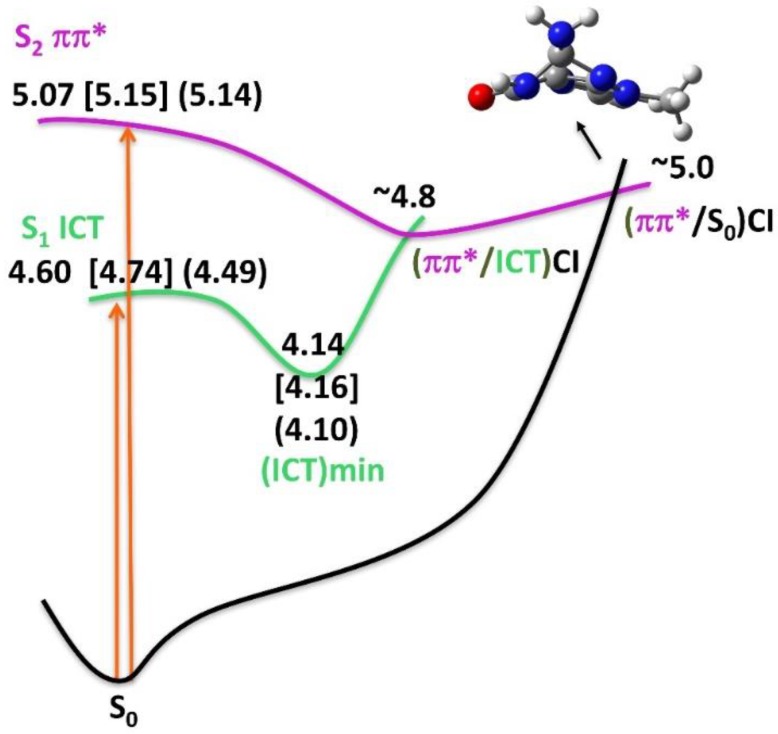
Main deactivation pathways along the ππ* (pink) and intramolecular charge transfer (ICT) (green) for 8vdG predicted by LR-PCM TD-CAM-B3LYP/6-31G(d) calculations in water. Energies are relative to the S_0_ (in eV) for 8vdG-*syn* + 4H_2_O, 8vdG-*anti* + 5H_2_O in brackets and 8vG in parenthesis.

The excited state PES were explored for the 8vdG conformers and 8vG along the bright S_1_ and S_2_ states, optimizing the relaxed geometries and looking for eventual deactivation paths (Table S6). The resulting picture is sketched in Figure 6. We focus our discussion mainly on the paths in H_2_O, for which the TR experimental data are available. 

Optimization of the S_1_ ICT state from the FC region leads to a stable minimum in the first excited PES, (ICT)-min, characterized by an increase of the C_8_-C_10_ bond distances, while C_4_-C_5_ and C_10_-C_11_ bond length decrease, together by small increase of the CT character (<0.02 a.u.) (Figure S7). For 8vdG, where the vinyl group is rotated with respect to the guanine at the GS-min geometry (-34anti/-17*syn*), a decrease of the dihedral angle at the (ICT)-min geometry is also observed (-8anti/-4 *syn*), leading to a more co-planar arrangement of the vinyl and purine moieties. The LR-PCM adiabatic and emission energies, at the (ICT)min, are 4.14–4.16 eV and ~3.7 eV (~335 nm), respectively. If solvent equilibration is considered via SS-PCM equilibrium single point calculations, the (ICT)min is further stabilized in ~0.14 eV and the emission energies peak at 3.5 eV (354 nm). Emission from (ICT)min would thus be characterized by a Stokes Shift of 0.8~1 eV (depending on the computational model and on the conformer considered. 

We have also computed the emission energies by SS-PCM calculations in the neq- limit. This approach has been profitably adopted in the past to study dynamical solvent shifts in fluorescence spectra [45] and to compute solvent reorganization energy [46]. Interestingly, solvent equilibration provides an energetic contribution of ~0.15 eV, a value fully consistent with the rapid red-shift during the first 1~2 ps, confirming our hypothesis that the ‘fast’ time constant in the fluorescence decay is related to solvation dynamics. 

An extensive exploration of the S_1_ PES does not allow finding any easily accessible crossing region with the GS. The only possible non-radiative deactivation route involves the ππ*/ICT crossing (described below) and is significantly higher in energy (+0.7 eV) than (ICT)min. Our calculations thus give full account of the strong and long-lived fluorescence of 8vdG.

When S_2_ ππ* state is instead optimized, a crossing with the lower lying ICT state is found (~4.8 eV), not far from the FC region. Subsequent S_1_ optimization ends up in the above-described (ICT)min. If the latter path is not followed, the system could deactivate to the ground state through a ππ*/S_0_ degeneracy region, which is rather close in energy to ππ*/ICT crossing (~5 eV) and more stable than S_2_ in the GS minimum. Reaching this funnel requires the out of plane distortion of the C_2_-NH_2_ group, similar to the well-described S_1_/S_0_ CI in guanine. This crossing region is 1.2~1.3 eV higher in energy than (ICT)min. Thereby, if the ππ* state absorbs at the FC either population of the stable excited ICT min or repopulation of the ground state are predicted. 

Since our calculations indicate the presence of a single stable emissive minimum (ICT)min, we can characterize the fluorescence anisotropies that would characterize the two different processes, that is, absorption in S_1_ → emission (ICT)min (mechanism 1) and absorption in S_2_ → S_1_ → emission (ICT)min (mechanism 2). Mechanism 1 (which does not imply any change in the absorbing and in emitting state) is characterized by an initial anisotropy of nearly 0.4. Mechanism 2 is mirrored by a smaller anisotropy (<0.2, see Table 4). 

Although, qualitatively, the main features of the excited PESs are common for the three systems (8vG, 8vdG-*syn* and 8vdG-anti) in H_2_O, some differences are evident when comparing these properties. The emission energy from (ICT)min is lower for the anti-conformer, suggesting that this species is characterized by red-shifted fluorescence with respect to the *syn* one. Analogously, 8vdG-anti-5H_2_O presents the largest Stokes shift, (1.05 eV) for the FC(ICT)-(ICT)min path compared to the *syn* conformer and 8vG (~0.8 eV). The latter result could be connected to the larger conformation rearrangement that planarization of δ induces in the sugar. The values of the main stationary points of FC(ππ*)-(ICT)min path are instead very similar for all the systems examined. 

For what concerns the calculated anisotropies, the only remarkable difference is the 0.17 value for the FC(ππ*)-(ICT)min path associated to the 8vdG-anti-5H_2_O conformer which is larger than the 0.11 computed for *syn* conformer and 8vG.

Geometry optimizations in MeCN provide a picture similar to that obtained in H_2_O. Interestingly, the computed emission energies are noticeably blue-shifted with respect to those obtained in H_2_O, in agreement with the experimental results. At the equilibrium level, the blue-shift between the same conformer is ≥0.1 eV. Furthermore, the relative stability of the *syn*-conformer, characterized by blue-shifted emission, increases in MeCN with respect to water, leading to an additional blue-shift with respect to water, almost recovering the experimental differences in the fluorescence maxima. The non-radiative deactivation path is similar to that described above in water but the energy gap between (ICT)-min and the degeneracy region with S_0_ is smaller, that is ~0.7 eV.

Finally, excited state optimizations in THF provide a picture similar to that depicted in the other solvents, with an additional 800 cm^−1^ blue-shift of the emission energy with respect to the latter solvent, in nice agreement with experimental results. Interestingly, the emission energies computed in THF and MeCN at the LR-PCM level are instead the same, confirming the importance of resorting to SS-PCM method to treat transitions with partial CT character. The computed radiative time constants are instead underestimated with respect to the experimental ones, likely due to the overestimation of the oscillator strength of S_1_, already highlighted when discussing the absorption spectra. The most stable crossing region with S_0_ is reached as in MeCN and in H_2_O and it is predicted to be less stable than (ICT)-min by ~0.6 eV.

## 4. Discussion

In this paper we report an extensive exploration of the photophysical behavior of a promising fluorescent DNA base analogue, 8vdG, in five solvents (THF, MeCN, EtOH, MeOH, H_2_O), with different polarity and hydrogen bonding ability. Our combined experimental/theoretical study gives a consistent picture of the absorption and emission spectra of 8vdG and show how a subtle interplay between intra-molecular and inter-molecular effects rules its optical properties in solution. 

The analysis of the experimental spectra shows that in all the solvent examined the first absorption band is composed of two transitions around 32,000 and 35,000 cm^−1^, that is, separated by about 0.4 eV, in excellent agreement with the calculations. The intensity of the S_2_–S_0_ transition is higher than that of S_1_–S_0_, contrary to our computational predictions. However, calculations show that the relative intensity of the two lowest bright states is strongly modulated by the mutual orientation of the vinyl moiety and the purine ring (δ dihedral), which govern the interaction between their π systems. In the ground electronic state at room temperature, the population of structures with significant deviation from a planar arrangement can be significant. Such structures exhibit a blue-shifted absorption spectrum and S_1_ and S_2_ have similar intensity. It is thus clear that only a fully vibronic treatment (well outside the scope of the present study), including the effect of low energy vibrational modes, can definitively assess how the interplay between the lowest energy excited states of 8vG govern its absorption spectrum. Our calculations also show that deviations from the planarity are also modulated by the substituent in 9-position and that also the coordination geometry of the solvent molecules is not anodyne. For example, in the minimum of *anti*-8vdG conformer, when also 5 water molecules are included in the computational model, δ is larger than 30°. In this scenario is likely that also the puckering of the deoxyribose sugar, can slightly affect the steric hindrances with the vinyl moiety and, therefore, the optical properties of 8vdG.

As previously noted [24], the S_0_ → S_1_ transition has a partial Gua→Vinyl CT character, which, interestingly, is also modulated by δ Our calculations show indeed that it increases for planar arrangement and that, as expected, the transition energy red-shifts when the polarity of the embedding medium increases. 

In summary, our analysis shows that solvent can have both direct and indirect, that is, mediated by the structural rearrangement induced in the solute, effects on the transition energy. Directly, by increasing the CT character of S_0_ → S_1_ transition. Indirectly, by affecting (i) the *syn*/*anti* equilibrium (ii) the puckering of the sugar (iii) the solute-solvent coordination geometry and, therefore, in non-easily predictably ways the value of δ. 

These considerations can give account of a seemingly counterintuitive experimental result, that is, an electronic transition with partial CT character that blue-shifts when going from THF to water and, at the same time, indicating that any conclusion issued only by analyzing the minima has to be considered with caution.

Experiments and calculations provide instead a less complex picture of the emission process, which confirms the previous gas phase analysis of Miller and coworkers [24]. Only a single minimum is present on S_1_, with significant CT character, which is separated by a sizeable energy barrier from the closest CI with S_0_. Excited state population on S_2_ is predicted to decay to such a minimum. On this ground, we can explain the high fluorescence yield of 8vdG and the low time-zero fluorescence anisotropy value (0.19) observed. Exciting 8vdG at 267 nm populates the second electronically excited state S_2_ but the fluorescence, on the other hand, emanates from the long-lived S_1_ CT state. The calculated Stokes shifts are consistent with the experimental ones, albeit the solvent dependence is less important. The increasing red-shift of the emission with increasing solvent polarity and hydrogen bonding ability are due to the clear CT character of the S_1_ minimum wherefrom the emission emanates.

In order to further corroborate this conclusion we tried to correlate our experimental results with some commonly used solvent descriptors. 

Our computational pictures is also consistent with the analysis of the fluorescence decays, which is clearly bi-exponential, with a fast time component on the ~1 ps time-scale and a slower one on the timescale of tens of picoseconds. The former is very likely related to solvation dynamics. The latter can be tentatively ascribed to a large-scale conformational rearrangement. As discussed above, we have a relatively free motion around δ in the ground state, and, conversely, the conformational space accessible to the sugar (and the coordinate water molecules) is relatively large. In the S_1_ minima, a planar arrangement around δ is strongly favored, restricting the conformational space available for the sugar. It is likely that this can also affect the puckering equilibrium in the sugar, while it is at the moment not possible to predict the consequences on the syn-anti equilibrium. 

In any case, such a large-amplitude motion can be expected to be much slower than solvation dynamics and could thus explain the picosecond spectral relaxation observed. Further time-resolved measurements in various solvents are needed in order to elucidate this subject.

There is no indication of any effective non-radiative deactivation channels, neither from experiment nor theory. Indeed, the measured excited state lifetimes are very long, about 3–4 ns. No strong solvent effects are observed but there is an indication of contributions from hydrogen bonding.

Using a simple Franck-Condon approximation, Miller and coworkers estimated the radiative lifetime of 8-vinylguanine (8vG) in aqueous solution to 1.5 ns [24]. Using the published value for the fluorescence quantum yield, Φ_F_ = 0.72 ± 0.3, [21] they deduced that the fluorescence lifetime in water should be around 1.1 ns. This is substantially shorter than what we observe for 8vdG in water (4.35 ns). Interestingly, they predicted that the excited state lifetime should diminish with decreasing solvent polarity, due to an accelerated radiationless decay. This radiationless decay channel involves a barrier-hindered crossing between the S_1_ ICT and S_2_ ππ* states and a subsequent conical intersection to the ground state. Our calculations show that this deactivation channel is active in all the three solvents examined and that the energy gap between the crossing region with S_0_ and the most stable S_1_ min, that is, (ICT)-min, increases in the order THF < MeCN << H_2_O. This indication is in line with the experimental lifetimes. We have therefore strong experimental and computational indications that the solvent polarity or polarizability are not the only or the dominant, factors determining the excited state dynamics of 8vdG. Hydrogen bonding is also contributing to the excited state deactivation. 

Our study therefore provides interesting insights on the possible use of 8vdG (and, likely, other vinyl-substituted purines) as fluorescent base analogue to be included in oligonucleotide. The dependence of the optical properties on the value of δ suggest that this probe could be extremely sensitive to very local conformational variation of DNA structures, for example involving a minor re-arrangement of its own sugar. As a consequence, this feature could make it more difficult to use this probe to monitor global conformational transitions, for example, increasing the local polarity experienced by the probe. On the other hand, this dependence could provide an additional and precious source of information. At the same time, the subtle dependence of the optical properties of 8vdG on the solvent features, that is, not only on the polarity but also on the solute-solvent Hydrogen Bonds, is particularly attracting, as well as the bi-exponential fluorescence decay, calling for additional time-resolved studies of this probe within DNA. 

## Figures and Tables

**Table 1:** Main photophysical parameters of 8vdG in five solvents.

	THF	MeCN	EtOH	MeOH	H_2_O
Absorption max (nm)	293	286	284	284	283
Absorption max (10^3^ cm^−1^)	34.1	34.9	35.2	35.2	35.4
Fluorescence max (nm)	370	376	380	384	396
Fluorescence max (10^3^ cm^−1^) ^a^	26.9	26.3	26.0	25.8	24.8
τ_F_ (at fluo-max) (ns)	2.07	2.73	3.10	3.17	4.35

^a^ Fluorescence spectra were scaled by a λ^2^ factor prior to the calculation of the peak wavenumbers.

**Table 2:** Fitted time constants of the fluorescence upconversion (FU) fluorescence decays. The uncertainties are typically about 2% but three digits are given in order for easier comparison.

	τ_1_	a_1_	τ_2_	a_2_	τ_3_	a_3_
330 nm	0.836	0.832	6.74	0.116	(4350)	0.0528
340 nm	1.09	0.810	19.0	0.101	(4350)	0.0890
350 nm	1.20	0.694	23.5	0.104	(4350)	0.203
360 nm	1.36	0.532	41.5	0.125	(4350)	0.343
370 nm	1.66	0.294	21.5	0.104	(4350)	0.601
380 nm	1.00	0.153	14.2	0.0890	(4350)	0.758
425 nm	0.486	−0.194	4.82	−0.219	(4350)	1.41
450 nm	1.62	−0.390	11.7	−0.141	(4350)	1.53
475 nm	2.52	−0.360	10.6	−0.105	(4350)	1.47
500 nm	0.935	−0.155	6.18	−0.248	(4350)	1.40
550 nm	2.40	0.232			(4350)	0.768

**Table 3:** Vertical Absorption Energies in eV [nm] and oscillator strengths for the different systems under study in water, acetonitrile and THF computed at the LR-PCM TD-CAM-B3LYP/6-31G(d) level of theory. In italics non-equilibrium SS-PCM TD-CAM-B3LYP/6-31G(d) energies.

		8vdG-*anti*		8vdG-*syn*		8vG	
H_2_O		(+5H_2_O)		(+4H_2_O)		(+5H_2_O)	
		ΔE	*f*	ΔE	*f*	ΔE	*f*
S_1_	π(G)-π*(V) [ICT]	4.74 [262] *4.70*	0.4855	4.60 [269] *4.54*	0.5100	4.49 [276] *4.45*	0.5164
S_2_	π(G)-π*(G) *[L_b_]*	5.15 [240] *5.22*	0.2702	5.07 [244] *5.15*	0.2693	5.14 [241] *5.22*	0.2858
MeCN							
		ΔE	*f*	ΔE	*f*	ΔE	*f*
S_1_	π(G)-π*(V) [ICT]	4.64 [267] *4.57*	0.5983	4.64 [267] *4.58*	0.6033	4.58 [270] *4.52*	0.5946
S_2_	π(G)-π*(G) *[L_b_]*	5.10 [243] *5.17*	0.1590	5.08 [244] *5.14*	0.1669	5.14 [241] *5.22*	0.1907
THF							
S_1_	π(G)-π*(V) [ICT]	4.64 [267] *4.60*	0.6266	4.64 [267] *4.60*	0.6315	4.58 [270] *4.53*	0.6214
S_2_	π(G)-π*(G) *[L_b_]*	5.09 [244] *5.14*	0.1409	5.07 [244] *5.12*	0.1568	5.13 [242] *5.20*	0.1813

**Table 4:** Emission energies at the (ICT)min, Stokes Shift eV and anisotropy (r) values for the different systems under study in H_2_O, MeCN and THF computed at the LR-PCM TD-CAM-B3LYP/6-31G(d) level of theory. Emission energies computed at the SS-PCM are in italics, NE superscript referring to non-equilibrium value, E to equilibrium ones). The computed radiative lifetimes (ns) are also reported the experimental values taken from Table S1 (transformed to eV) are also shown.

	8vdG-*anti*				8vdG-*syn*				8vG			8vdG Exp
Minima	ΔE	Stokes	R	${}^{Theor}\tau_{R}$	ΔE	Stokes	r	${}^{Theor}\tau_{R}$	ΔE	Stokes	r	Stokes
H_2_O												
FC(ICT)- (ICT)min	3.69 *^NE^3.64* *^E^3.48*	1.05	0.37	0.53 *0.86*	3.73 *^NE^3.68* *^E^3.53*	0.86	0.39	0.49 *0.80*	3.71 *^NE^3.66* *^E^3.49*	0.78	0.40	
FC(ππ*)- (ICT)min		1.46	0.17			1.34	0.11			1.43	0.11	1.44
MeCN												
FC(ICT)- (ICT)min	3.76 *^NE^3.71* *^E^3.57*	0.88	0.36	0.43 *0.70*	3.81 *^NE^3.77* *^E^3.64*	0.83	0.40	0.40 *0.64*	3.78 *^NE^3.73* *^E^3.57*	0.80	0.40	
FC(ππ*)- (ICT)min		1.34	0.08			1.28	0.06			1.36	0.11	1.13
THF												
FC(ICT)- (ICT)min	3.76 *^NE^3.75* *^E^3.66*	0.88	0.40	0.40 *0.64*	3.80 *^NE^3.81* *^E^3.73*	0.84	0.40	0.37 *0.58*	3.78 *^NE^3.77* *^E^3.67*	0.80	0.40	
FC(ππ*)- (ICT)min		1.33	0.04			1.27	0.04			1.35	0.09	0.98

## Supplementary Materials

The following are available online at https://www.mdpi.com/1420-3049/25/4/824/s1. Absorption spectra on an energy scale of 8vdG in the five solvents water, methanol, ethanol, acetonitrile and tetrahydrofuran. Second derivative of the absorption spectra in the five solvents. Decomposition of the absorption spectra in the five solvents using two lognormal functions. Table with resulting photo physical parameters in the five solvents, absorption and fluorescence peak frequencies, fluorescence lifetimes and Stokes shifts as well as the solvent polarities and hydrogen-bond abilities. Fitted time resolved fluorescence spectra of 8vdG in water. The time-evolution of the mean frequency of 8vdG in water between 0 and 90 ps. Ground State Conformational Analysis in water and acetonitrile, ground syn/anti stability, rotation energies (kcal/mol) of the vinyl group, optimized geometries for 8vdG-syn and 8vdG-anti. Vertical absorption energies. Excited state density differences (S_1_-S_0_). Excited state conformational analysis in water, acetonitrile and tetrahydrofuran, potential energy surface in water and acetonitrile, excited state syn/anti stability, excited state (S_1_) geometry.
