# Supplementary material for: Excited State Dynamics of 8-Vinyldeoxyguanosine in Aqueous Solution Studied by Time-Resolved Fluorescence Spectroscopy and Quantum Mechanical Calculations
Source: Molecules. 2020 Feb 13;25(4):824. doi: 10.3390/molecules25040824 (PMC7071107; doi:10.3390/molecules25040824)
Supplement: Supplementary file 1 [file molecules-25-00824-s001.pdf]

molecules

MDPI

Supplementary Materials

Excited State Dynamics of 8-Vinyldeoxyguanosine In Aqueous Solution Studied by Time-Resolved Fluorescence Spectroscopy and Quantum Mechanical Calculations

Lara Martinez-Fernandez [1], Thomas Gustavsson [2,*], Ulf Diederichsen [3] and Roberto Improta [4,*]

[1] Departamento de Química, Facultad de Ciencias and IADCHEM (Institute for Advanced Research in Chemistry) Universidad Autónoma de Madrid, Cantoblanco, 28049 Madrid, Spain; lara.martinez@uam.es
[2] Université Paris-Saclay, CEA, CNRS, LIDYL, 91191 Gif-sur-Yvette, France
[3] Univ Goettingen, Inst Organ & Biomol Chem, Tammannstr 2, D-37077 Goettingen, Germany; udieder@gwdg.de
[4] Istituto di Biostrutture e Bioimmagini, CNR, Via Mezzocannone 16, I-80134 Napoli, Italy
* Correspondence: thomas.gustavsson@cea.fr (T.G.); robimp@unina.it (R.I.).

Abstract: The fluorescent base guanine analog, 8-vinyl-deoxyguanosine (8vdG), is studied in solution using a combination of optical spectroscopies, notably femtosecond fluorescence upconversion and quantum chemical calculations, based on time-dependent density functional theory (TD-DFT) and including solvent effect by using a mixed discrete-continuum model. In all investigated solvents, the fluorescence is very long lived (3–4 ns), emanating from a stable excited state minimum with pronounced intramolecular charge-transfer character. The main non-radiative decay channel features a sizeable energy barrier and it is affected by the polarity and the H-bonding properties of the solvent. Calculations provide a picture of dynamical solvation effects fully consistent with the experimental results and show that the photophysical properties of 8vdG are modulated by the orientation of the vinyl group with respect to the purine ring, which in turn depends on the solvent. These findings may have importance for the understanding of the fluorescence properties of 8vdG when incorporated in a DNA helix.

Keywords: DNA; solvent effect; TD-DFT; fluorescent probe

Absorption spectra in different solvents

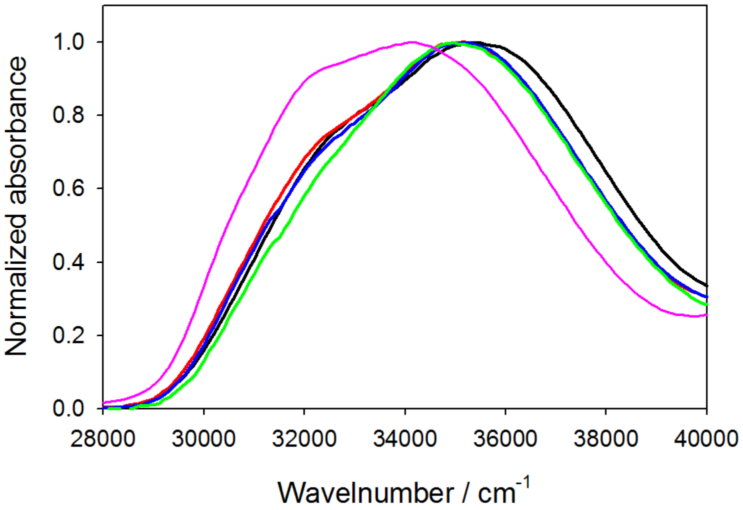

Figure S1. Normalized absorption spectra on a wavenumber scale of 8vdG in water (black), methanol (red), ethanol (blue), acetonitrile (green) and THF (pink).

Table S1. Additional photophysical parameters of 8vdG in different solvents.

	**THF**	**MeCN**	**EtOH**	**MeOH**	**H_2O**
Absorption max (10^3 cm^{-1})	34.1	34.9	35.2	35.2	35.4
Absorption max 1 (10^3 cm^{-1}) [a]	31.5	31.9	31.8	31.8	32.3
Absorption max 2 (10^3 cm^{-1}) [a]	34.8	35.4	35.5	35.5	36.1
Fluorescence max (10^3 cm^{-1}) [b]	26.9	26.3	26.0	25.8	24.8
Stokes shift 1 $\Delta\sigma$ (10^3 cm^{-1})	4.6	5.6	5.8	6.0	7.5
Stokes shift 2 $\Delta\sigma$ (10^3 cm^{-1})	7.9	9.1	9.5	9.7	11.6
Solvent polarity [c]	7.58	35.96	25.3	33.1	77.97
Solvent H-bond donor [d]	0	0.19	0.83	0.93	1.17

[a] Decomposing the experimental absorption spectrum using a sum of two lognormal functions.

[b] Fluorescence spectra were scaled by a λ^2 factor prior to the calculation of the peak wavenumbers.

[c] From Riddick et al. [1] .

[d] From Marcus et al. [2].

Second derivative of the absorption spectra

The absorption spectra shown in Figure S1, were derived numerically in two steps. A first derivation to obtain the 1st deriviative which subsequently was derived a second time in order to obtain the 2nd deriative. In the figure below, the 2nd derivative was also smoothed over five times to obtain a less noisy curve.

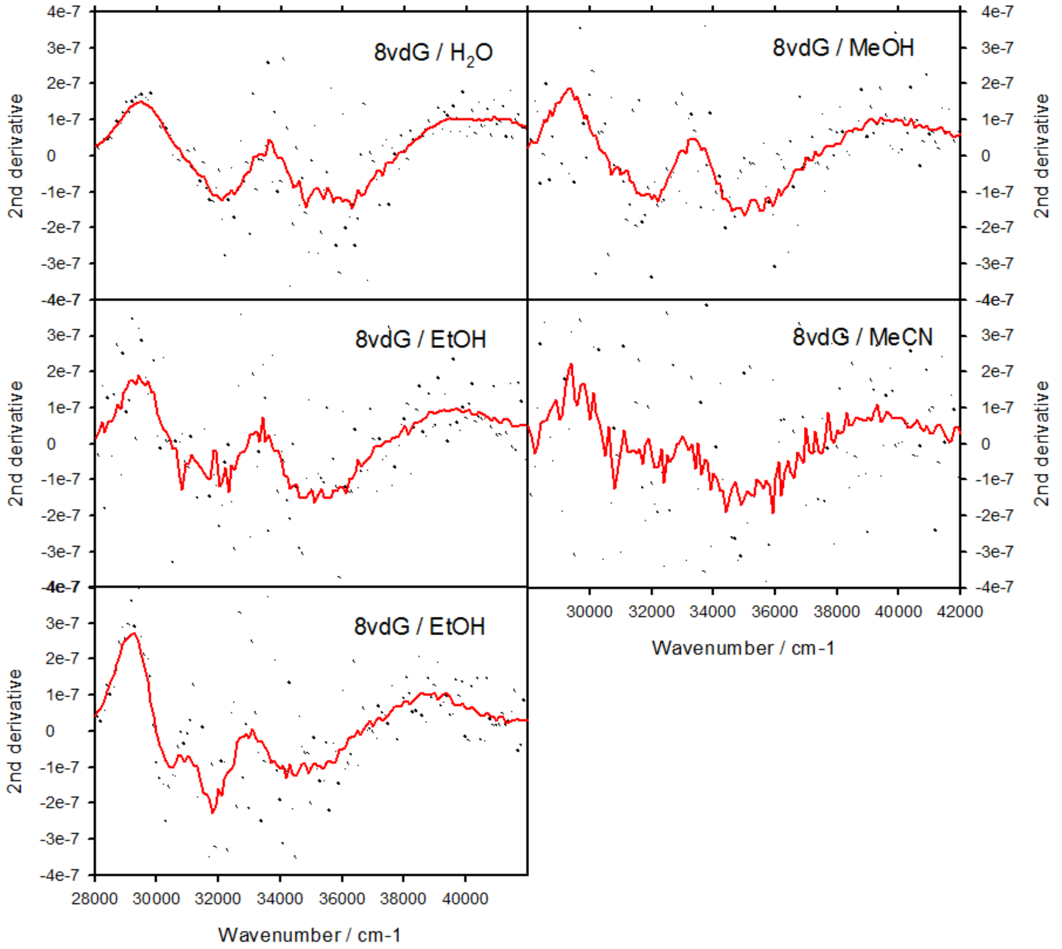

Figure S2. Second derivatives of the absorption spectra of 8vdG Raw data (black points) and 5 point smoothed data (red lines).

Decomposition of the absorption spectra using lognormal functions

As described in the main text, the absorption spectra were model-fitted by a sum of two simplified lognormal functions

$$A(\nu) = A_1 exp\left(-\beta_1^2\left[ln\frac{\nu - a_1}{\beta_1}\right]^2\right) + A_2 exp\left(-\beta_{21}^2\left[ln\frac{\nu - a_2}{\beta_{21}}\right]^2\right)$$

The peak frequency is given by

$$\nu_{peak} = a + b$$

and the resulting values are given in Table S1.

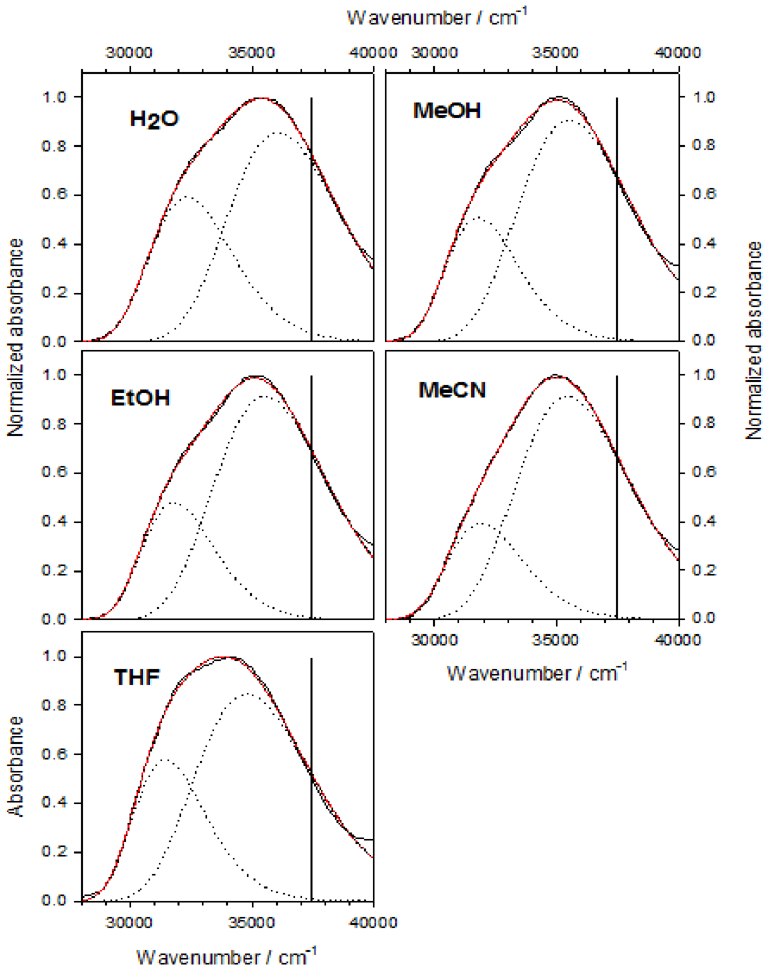

Figure S3. Decomposition of the absorption spectra of 8vdG in water (black), methanol (red), ethanol (blue) and acetonitrile (green) using a sum of two lognormal functions. The laser excitation is indicated by a black vertical line.

It should be noted that this specific model is an arbitrary choice, motivated by getting the best numerical fit so the results must be taken with much caution. For example, the peak of the experimental absorption spectrum red-shifts by only 1 nm (200 cm^{-1}) going from H_2O to MeOH while the fitted high-energy peak red-shifts by ~600 cm^{-1}. It should be noted that the fitted high-energy peaks are significantly blue-shifted with regards to the observed maximum in all solvents. It is difficult to estimate the uncertainties in the fitted peak values, but they extend to several hundreds of cm^{-1}. Other model functions were also used, for example the sum of two Gaussians, but without achieving the same level of fitting quality.

Fitted time resolved fluorescence spectra

To characterise the reconstructed time-resolved fluorescence spectra they were fitted with a simplified log-normal function [3].

$$I(\nu) = I_0 exp\left(-\beta^2 \left[ln\frac{\nu - a}{\beta}\right]^2\right)$$

This simplified log-normal function allows the easy calculation of several important spectral parameters. In particular, the mean frequency is given by the eq.

$$\nu_{mean} = a + b \cdot exp\left(\frac{3}{4\beta^2}\right)$$

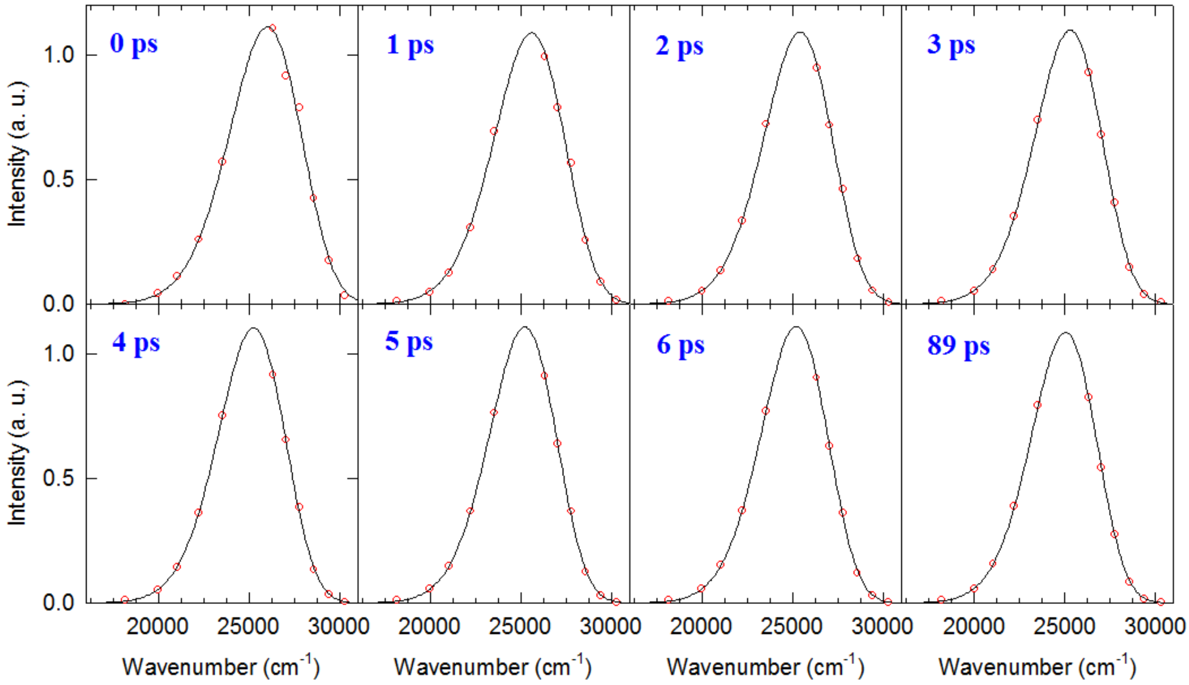

Figure S4. Reconstructed time resolved fluorescence spectra and corresponding lognormal fits at chosen delay times.

Ground State Conformational Analysis

Table S2. Syn/Anti stability computed at the PCM-CAM-B3LYP level of theory and with the specified basis set.

H_2O	8vdG-syn+4H_2O	H_2O		8vdG-anti+5H_2O	ΔE eV (kcal/mol)
**6-31G(d)**	-1346.14756	-76.38674	-1422.53431	-1422.54183	-0.20 (4.72)
**6-31+G(d,p)**	-1346.31054	-76.41408	-1422.72463	-1422.72526	-0.02 (-0.39)
**6-311+G(2d,2p)**	-1346.68290	-76.44098	-1423.12389	-1423.12373	0.00 (0.10)
**MeCN**	8vdG-syn			8vdG-anti	ΔE eV (kcal/mol)
**6-31G(d)**	-1040.51744			-1040.50925	0.22 (5.14)
**6-31+G(d,p)**	-1040.60307			-1040.59719	0.16 (3.69)
**6-311+G(2d,2p)**	-1040.87258			-1040.86639	0.17 (3.88)

Table S3. Rotation energies (kcal/mol) of the vinyl group (in S_0) in WAT and MeCN for 8vdG and 8vG at the PCM-CAM-B3LYP/6-31G(d) level of theory. Energies computed by single point calculations with larger basis set 6-311+G(2d,2p) are shown in parenthesis.

H_2O	S_0 min	45°	90°
8vdG-syn+4H_2O	0	0.80 (0.60)	3.50 (2.99)
8vdG-anti+5H_2O	0	0.18 (0.04)	3.51 (2.63)
8vG	0	1.47 (1.21)	4.57 (4.01)
**MeCN**			
8vdG-syn	0	1.04 (0.73)	3.75 (3.13)
8vdG-anti	0	1.48 (1.24)	7.39 (6.22)
8vG	0	1.86 (1.50)	5.07 (4.42)

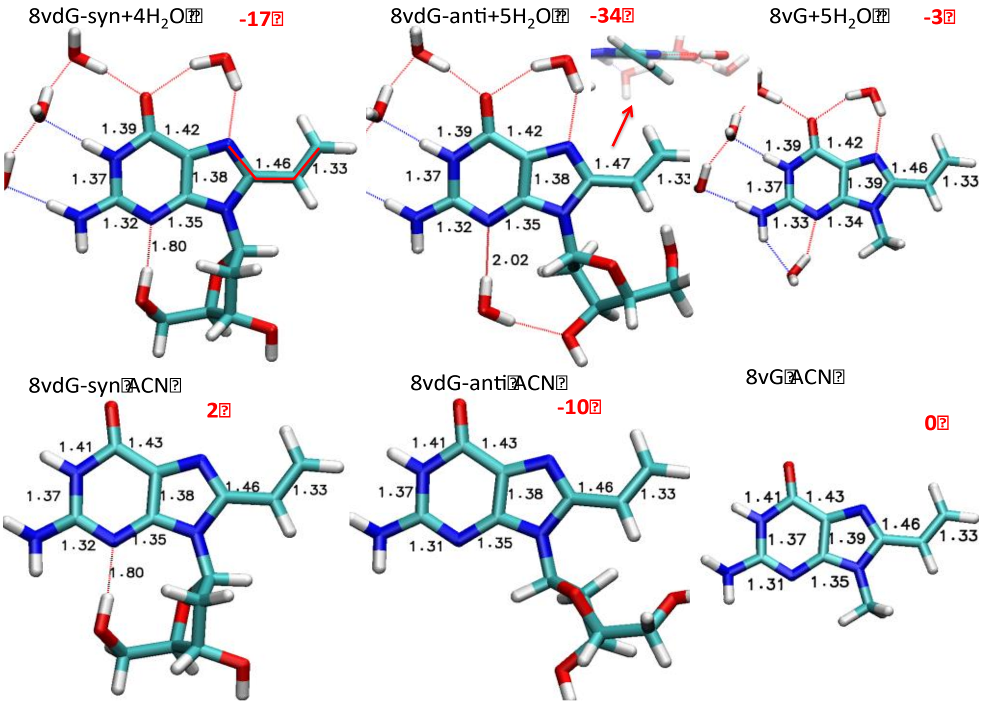

Figure S5. Optimized ground (S_0) minima for 8vdG-syn and 8vdG-anti in WAT and MeCN. Dihedral angle in red, dC_{11}-C_{10}-C_8-N_7, in degrees. PCM/CAM-B3LYP/6-31G(d).

Table S4. Vertical Absorption Energies in eV [nm] and oscillator strengths for 8vG under water and acetonitrile computed at the LR-PCM TD-CAM-B3LYP/6-31G(d) level of theory for different values of the δ dihedral. Dipole moments are specified in parenthesis. *[La] #[Lb].

		8vG		8vG		8vG	
**H_2O (+5H_2O)**		**(minimum) (6.4674)**		**(45°) (6.5063)**		**(90°) (6.6555)**	
		**ΔE**	***f***	**ΔE**	***f***	**ΔE**	**Ƒ**
S_1	π(G)-π*(V) [ICT]	4.49 [276]	0.5164 (10.7839)	4.80 [258]	0.4267 (10.5530)	5.58 [222]	0.5435 (6.2986)
S_2	π(G)-π*(G) *[L_b]*	5.14 [241]	0.2858 (6.1597)	*5.19 [239]	0.3509 (6.5101)	*5.06 [245]	0.1736 (8.7458)
S_6	π(G)-π*(G) *[L_a]*	6.32 [196]	0.4388	#6.50 [190]	0.4400	#6.73 [184]	0.3299
MeCN							
		**(minimum) (8.8600)**		**(45°) (8.8253)**		**(90°) (8.8588)**	
		**ΔE**	***f***	**ΔE**	***f***	**ΔE**	**Ƒ**
S_1	π(G)-π*(V) [ICT]	4.58 [270]	0.5946 (13.1027)	4.93 [251]	0.5678 (13.2511)	5.60 [221]	0.5328 (9.1274)
S_2	π(G)-π*(G) *[L_b]*	5.14 [241]	0.1907 (8.0968)	*5.18 [239]	0.1807 (8.4656)	*5.24 [236]	0.1565 (9.2811)
S_6	π(G)-π*(G) *[L_a]*	6.42 [193]	0.2931	#6.63 [186]	0.2637	#S_8 6.96	0.5022

Table S5. Vertical Absorption Energies in eV [nm] and oscillator strengths for 9methylguanine in water and acetonitrile computed at the LR-PCM TD-CAM-B3LYP/6-31G(d) level of theory. Dipole moments are specified in parenthesis.

		**G**	
**H_2O (+5H_2O)**		**(7.0638)**	
		**ΔE**	***f***
S_1	π(G)-π*(G) *[L_a]*	5.09<br>[244]	0.1661<br>(9.1668)
S_2	π(G)-π*(G) *[L_b]*	5.67<br>[219]	0.4115<br>(6.5636)
MeCN		(9.1003)	
S_1	π(G)-π*(G) *[L_a]*	5.27<br>[235]	0.1626<br>(9.4757)
S_2	π(G)-π*(G) *[L_b]*	5.69<br>[218]	0.3816<br>(9.4260)

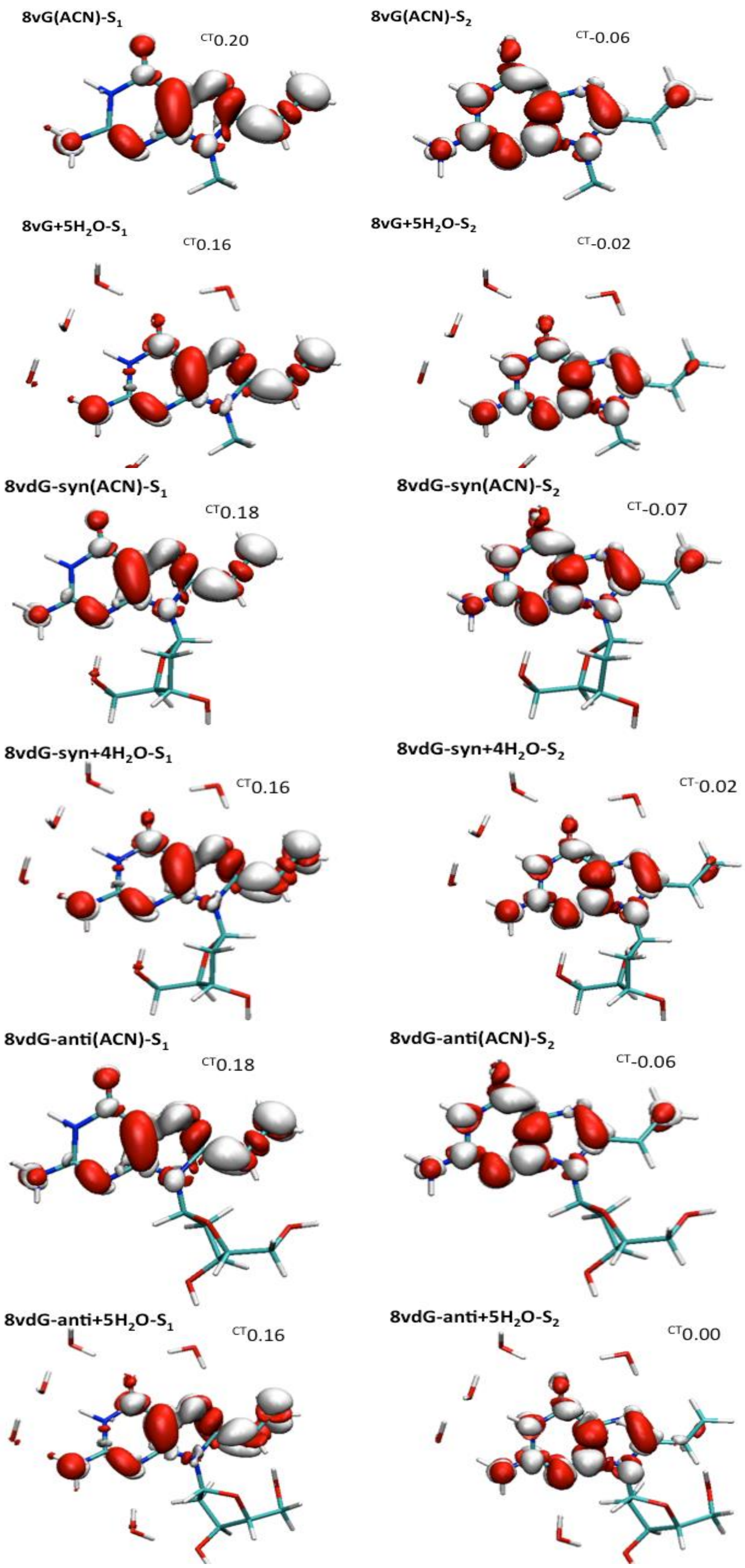

Figure S6. Excited state density difference (S_1-S_0) and charge transfer character (a.u.) between the G and vinyl moieties computed at PCM/TD-CAM-B3LYP/6-31G(d) level of theory.

Excited state Potential Energy Surface

Table S6. Potential Energy Surface in WAT and MeCN for 8vdG and in WAT, MeCN and THF. PCM/TD-CAM-B3LYP/6-31G(d).

**H_2O**	**FC-ICT**	**FC-pp***	**(ICT)min**	**ππ*/ICT**	**ππ*/S_0**
8vdG-syn+4H_2O	4.60	5.07	4.14	4.81	*5.35*
8vdG-anti+5H_2O	4.74	5.15	4.16	4.84	*5.31*
8vG	4.49	5.14	4.10	4.83	*5.30*
**MeCN**					
8vdG-syn	4.64	5.08	4.21	4.76	*4.81*
8vdG-anti	4.64	5.10	4.18	4.71	*4.84*
8vG	4.58	5.14	4.17	4.80	*4.86*
**THF**					
8vG	4.58	5.13	4.17	4.74	*4.60*

Table S7. Syn/Anti stability computed at the PCM-CAM-B3LYP level of theory and with the specified basis set in the Excited State minimum.

**H_2O**	**8vdG-syn+4H_2O**	**H_2O**		**8vdG-anti+5H_2O**	**ΔE eV (kcal/mol)**
**6-31G(d)**	-1345.99529	-76.38674	-1422.38204	-1422.38905	-0.19 (-4.40)
**6-31+G(d,p)**	-1346.16442	-76.41408	-1422.57851	-1422.57860	0.00 (-0.06)
**MeCN**	8vdG-syn			8vdG-anti	ΔE eV (kcal/mol)
**6-31G(d)**	-1040.36278			- 1040.35576	0.19 (4.40)
**6-31+G(d,p)**	-1040.45630			-1040.45152	0.13 (3.00)

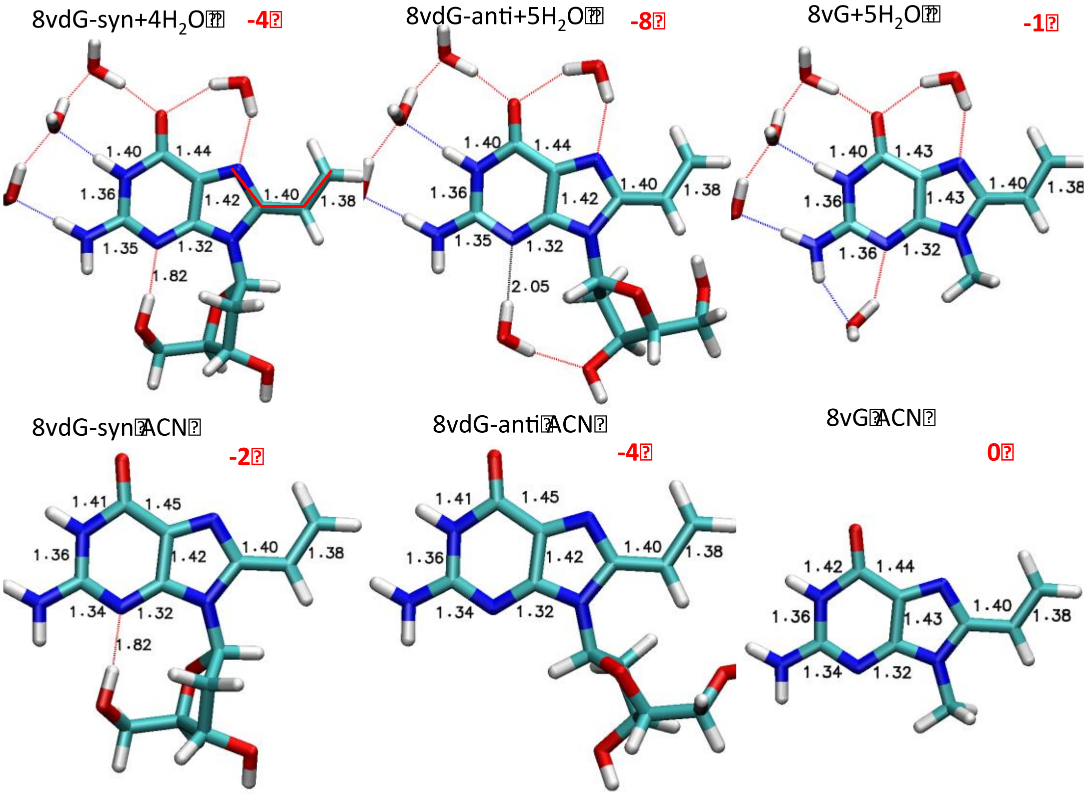

Figure S7. Excited state (S_1) minima for 8vdG-syn and 8vdG-anti in WAT and MeCN. Dihedral angle in red, dC_{11}-C_{10}-C_8-N_7, in degrees. PCM/TD-CAM-B3LYP/6-31G(d).

Full reference 39 in the manuscript

M. J. Frisch, G. W. Trucks, H. B. Schlegel, G. E. Scuseria, M. A. Robb, J. R. Cheeseman, G. Scalmani, V. Barone, B. Mennucci, G. A. Petersson, H. Nakatsuji, M. Caricato, X. Li, H. P. Hratchian, A. F. Izmaylov, J. Bloino, G. Zheng, J. L. Sonnenberg, M. Hada, M. Ehara, K. Toyota, R. Fukuda, J. Hasegawa, M. Ishida, T. Nakajima, Y. Honda, O. Kitao, H. Nakai, T. Vreven, J. A. Montgomery, Jr., J. E. Peralta, F. Ogliaro, M. Bearpark, J. J. Heyd, E. Brothers, K. N. Kudin, V. N. Staroverov, R. Kobayashi, J. Normand, K. Raghavachari, A. Rendell, J. C. Burant, S. S. Iyengar, J. Tomasi, M. Cossi, N. Rega, J. M. Millam, M. Klene, J. E. Knox, J. B. Cross, V. Bakken, C. Adamo, J. Jaramillo, R. Gomperts, R. E. Stratmann, O. Yazyev, A. J. Austin, R. Cammi, C. Pomelli, J. W. Ochterski, R. L. Martin, K. Morokuma, V. G. Zakrzewski, G. A. Voth, P. Salvador, J. J. Dannenberg, S. Dapprich, A. D. Daniels, Ö. Farkas, J. B. Foresman, J. V. Ortiz, J. Cioslowski, and D. J. Fox, *Gaussian 09* (Gaussian, Inc., Wallingford CT, 2009).

References

1. Riddick, J. A.; Bunger, W. B.; Sakano, T. K. *Organic Solvents, Physical properties and methods of purification*. Wiley Interscience: New York, 1986; Vol. II.
2. Marcus, Y.; Kamlet, M. J.; Taft, R. W. Linear solvation energy relationships. Standard molar Gibbs free energies and enthalpies of ions from water into nonaqueous solvents. *J. Phys. Chem.* **1988,** *92*, 3613-3622.
3. Gustavsson, T.; Cassara, L.; Gulbinas, V.; Gurzadyan, G.; Mialocq, J.-C.; Pommeret, S.; Sorgius, M.; van der Meulen, P. Femtosecond Spectroscopic Study of Relaxation Processes of Three Amino-Substituted Coumarin Dyes in Methanol and Dimethylsulfoxide. *J. Phys. Chem. A* **1998,** *102*, 4229-4245.
